# Endoplasmic Reticulum Stress Is Involved in Muscular Pathogenesis in Idiopathic Inflammatory Myopathies

**DOI:** 10.3389/fcell.2022.791986

**Published:** 2022-02-14

**Authors:** Xue Ma, Hua-Jie Gao, Qing Zhang, Meng-Ge Yang, Zhua-Jin Bi, Su-Qiong Ji, Yue Li, Li Xu, Bi-Tao Bu

**Affiliations:** Department of Neurology, Tongji Hospital, Tongji Medical College, Huazhong University of Science and Technology, Wuhan, China

**Keywords:** idiopathic inflammatory myopathies, endoplasmic reticulum stress, immune-mediated necrotizing myopathy, glucose-regulated protein 78 (GRP78)/BiP, skeletal muscle dysfunction

## Abstract

**Objectives:** Endoplasmic reticulum (ER) stress plays pivotal roles in the regulation of skeletal muscle damage and dysfunction in multiple disease conditions. We postulate the activation of ER stress in idiopathic inflammatory myopathies (IIM).

**Methods:** Thirty-seven patients with immune-mediated necrotizing myopathy (IMNM), 21 patients with dermatomyositis (DM), 6 patients with anti-synthetase syndrome (ASS), and 10 controls were enrolled. The expression of ER stress-induced autophagy pathway was detected using histological sections, Western blot, and real-time quantitative Polymerase Chain Reaction.

**Results:** ER stress-induced autophagy pathway was activated in biopsied muscle of patients with IMNM, DM, and ASS. The ER chaperone protein, glucose-regulated protein 78 (GRP78)/BiP expression in skeletal muscle correlated with autophagy, myofiber atrophy, myonecrosis, myoregeneration, and disease activity in IMNM.

**Conclusion:** ER stress was involved in patients with IIM and correlates with disease activity in IMNM. ER stress response may be responsible for skeletal muscle damage and repair in IIM.

## Introduction

Idiopathic inflammatory myopathies (IIM) is currently classified into four major subtypes: dermatomyositis (DM), immune-mediated necrotizing myopathy (IMNM), anti-synthetase syndrome (ASS), and sporadic inclusion body myositis (sIBM) based on clinicoseropathological features, myositis-specific antibodies, and transcriptomic signatures (Tanboon et al., 2020). Though they all share an immune-mediated physiopathologic mechanism, the pathogenesis likely differs among the subgroups, given their distinct serologic and clinicopathologic characteristics (Tanboon et al., 2020). IMNM has been only relatively recently recognized, and the molecular mechanisms underlying skeletal muscle dysfunction remain obscure (Allenbach, Mammen et al., 2018). The key immunological mechanisms underlying attack on muscle, including anti-myositis autoantibodies (Arouche-Delaperche et al., 2017) and complement (Bergua et al., 2019), are pathogenic toward muscle in IMNM. However, some patients with IMNM had poor response to powerful immunosuppressive therapies (Mecoli et al., 2017; Li et al., 2021), suggesting that non-immune mediators also play important roles in skeletal muscle dysfunction in the disease condition.

The endoplasmic reticulum (ER) stress three sensors, inositol-requiring enzyme 1 (IRE1), protein kinase R-like ER kinase (PERK), and activating transcription factor 6 (ATF6), regulate unfolded protein response (UPR) signaling by recognizing the unfolded proteins (Salminen et al., 2009). These three sensors normally integrate with the chaperone glucose-regulated protein 78 (GRP78)/BiP in the ER lumen. BiP triggers UPR by dissociating and accelerating the accumulated proteins to fold when unfolded proteins accumulate over much in the ER lumen. UPR signaling protects cells or induces cell death by regulating autophagy under extreme conditions (Song et al., 2017). A previous study revealed chaperone-assisted selective autophagy in IMNM (Fischer et al., 2019; Girolamo et al., 2019). However, whether the activation of ER stress-induced autophagy pathways is involved in IMNM and other IIM subtypes remains elusive. ER stress is likely to play a role in the pathophysiology of IIM (Nagaraju et al., 2005; Fischer et al., 2019) and may be responsible for muscle damage, but it may paradoxically assist in muscle restoration (Bohnert et al., 2018). Therefore, the role of ER stress within IIM muscle appears complex and clearly needs further exploration. Moreover, research investigating expression of ER stress-associated proteins in IIM has focused on DM, polymyositis and sIBM. These findings may, however, not be readily extrapolated to the condition of IMNM.

## Materials and Methods

### Patient Selection

Muscle tissue and clinical data were obtained from the Department of Neurology at Tongji Hospital from January 1, 2013 to September 1, 2021. The diagnosis of IMNM and DM was made according to the European Neuromuscular Centre International Workshop on Idiopathic Inflammatory Myopathies (Hoogendijk et al., 2004; Allenbach, Mammen et al., 2018). Patients with myositis were classified as ASS if tested positive for anti-synthetase autoantibodies (Pinal-Fernandez et al., 2017). Subjects, presenting with diffuse myalgia, weakness, or unexplained creatine phosphokinase (CK) elevations, but which lacked any myopathic features, serves as controls. The exclusion criteria included patients with muscular dystrophy, metabolic myopathy, congenital myopathy, cancer-associated myositis, other connective tissue disease overlap myositis, and infection-, drug-, or toxin-induced myopathies. Patients with insufficient clinical data, and thawed, damaged, or insufficient tissue for sectioning were also excluded. The levels of serum CK and lactic dehydrogenase (LDH) were obtained at the time of muscle biopsy. Muscle strength was evaluated based on the manual muscle testing (MMT)-8 scores (scale 0–80) at the time of diagnosis (Rider et al., 2010). The study was approved by the Ethics Committee of Tongji Hospital (IRB ID: TJ-C20121221), and written informed consent was obtained from all subjects.

All serums from included subjects were tested for myositis-specific antibodies (MSAs) and myositis-associated antibodies (MAAs). The following MSAs and MAAs were assessed using two commercial semiquantitative line blot assays (D-Tek, Germany; Euroline, Germany): IMNM-specific-antibodies [anti-signal recognition particle (SRP) and anti-3-hydroxy-3-methylglutaryl-coenzyme a reductase (HMGCR)], DM-associated antibodies [anti-mitochondrial (Mi) 2α and β, anti-transcriptional intermediary factor 1γ (TIF1γ), anti-melanoma differentiation-associated protein 5 (MDA5), anti-nuclear matrix protein 2 (NXP2), and anti-small ubiquitin-like modifier activating enzyme 1 (SAE1)], anti-synthetase antibodies [anti-histidyl-tRNA synthetase (Jo-1), anti-alanyl-tRNA synthetase (PL-12), anti-glycyl-tRNA synthetase (EJ), anti-isoleucyl-tRNA synthetase (OJ), and anti-threonyl-tRNA synthetase (PL-7)], IBMs-specific antibodies (anti-cN-1A), anti-Ku, anti-PMScl100, anti-PMScl75, and anti-Ro52 antibodies (Cavazzana et al., 2016).

### Muscle Biopsy and Immunohistochemistry

All enrolled patients performed skeletal muscle biopsy for diagnosis. Serial 7-μm-thick frozen sections were stained using routine methods including hematoxylin–eosin, modified Gomori’s trichrome, acid phosphatase, NADH-tetrazolium reductase, Sudan black, cytochrome C oxidase, succinate dehydrogenase, periodic acid-Schiff, oil red O, and myosin ATPase. The following primary antibodies were used for immunohistochemical staining: BiP (1:200, 11587-1-AP, Proteintech), lysosomal-associated membrane protein 2 (LAMP2) (1:200, H4B4, Developmental Studies Hybridoma Bank), p62 (sequestosome 1) (1:400, 18420-1-AP, Proteintech), LC3 (1:200, A19665, ABclonal), muscle RING Finger protein-1 (MuRF1) (1:50, ab172479, Abcam), muscle atrophy F-box (MAFbx)/Atrogin-1 (1:50, bs-2591R, Bioss Biotech), dystrophin (1:30, NCL-DYS1, Leica), and neural cell adhesion molecule/CD56 (1:50, ab6123, Abcam). Then horseradish peroxidase-labeled anti-rabbit, or anti-mouse secondary IgG antibodies (SV0004, Boster) or appropriate Alexa Fluor-conjugated secondary antibody (Invitrogen) were applied according to the protocol of the manufacturer.

### Western Blot

Muscle tissue were dissected and homogenized in cell lysis buffer (Servicebio, China) added with phosphatase inhibitors (Promotor, China). The lysates were centrifuged at 12,000 rpm for 15 min at 4°C. Supernatant was collected, and protein concentration was determined using bicinchoninic acid assays. Total protein (20–40 μg) was electrophoresed on 8%–10% sodium dodecyl sulfate-polyacrylamide gels and transferred to nitrocellulose filter membranes. Then the membranes were probed with primary antibodies: GRP78/BiP (1:1,000, 3,177, Cell Signaling Technology), p62 (1:1,000, 18420-1-AP, Proteintech), ATG12 (1:1,000, 4,180, Cell Signaling Technology), Beclin-1 (1:1,000, 3,495, Cell Signaling Technology), Phospho-SAPK/JNK (Thr183/Tyr185) (P-JNK) (1:1,000, 4,668, Cell Signaling Technology), JNK1 (1:1,000, 3,708, Cell Signaling Technology), Phospho-eIF2α (P-eIF2α) (1:1,000, 3,398, Cell Signaling Technology), eIF2α (1:1,000, 5,324, Cell Signaling Technology), and glyceraldehyde-3-phosphate dehydrogenase **(**GAPDH) (1: 5,000, 60004-1-Ig, Proteintech). Then the membranes were incubated with horseradish peroxidase-labeled anti-rabbit, or anti-mouse secondary antibody (1:5,000, Cell Signaling Technology). Target protein bands were visualized with chemiluminescence reagents and assessed using Fiji software (NIH).

### Reverse Transcription Quantitative Polymerase Chain Reaction

Total RNA was extracted from muscle specimens using Trizol (Invitrogen). Afterward, cDNA was synthesized using PrimeScript™ RT Master Mix (Perfect Real Time) (Takara). Following this, primers for GAPDH, autophagy related 5 (ATG5), autophagy-related 2B (ATG2B), phosphatase and tensin homolog (PTEN)-induced putative kinase 1 (PINK1), C/EBP Homologous Protein (CHOP), BiP, sequestosome 1 (SQSTM1**)**, ER degradation-enhancing alpha-mannosidase-like protein 1 **(**EDEM1), BCL2-interacting protein 3 (BINP3), and X-box-binding protein 1 (XBP1) were analyzed by RT-qPCR with SYBR-Green assays (Yeasen). GAPDH was used for internal reference. RT-qPCR reactions were performed with BioRad CFX Connect system. The primer sequences are displayed in Supplementary Table S1. The fold changes of target genes were calculated on the basis of the 2−ΔΔCT method (Livak and Schmittgen, 2001).

### Transmission Electron Microscopy

The ultrastructural analysis of the muscle specimens was performed after the samples were fixated in 2.5% glutaraldehyde for 48 h at 4°C, post-fixated in 1% osmium tetroxide, and embedded in Araldite. Then ultrathin sections were stained with both uranyl acetate and lead citrate to be analyzed using the Hitachi TEM system at 80.0 KV.

### Statistical Analysis

We randomly selected five fields of ×200-fold magnification in each section. Then the total number of muscle fibers was manually counted, and the average percentages of target antibody-positive myofibers, necrotic myofibers, and regenerating myofibers from each section were calculated. Feret diameter of each muscle fiber outlined by dystrophin2 immunostaining was analyzed using Fiji software (Briguet et al., 2004). The variability coefficient was calculated as follows: variability coefficient = 1,000 ✕ standard deviation of muscle fiber minimal diameters/mean muscle fiber minimal diameter (Arouche-Delaperche et al., 2017). Necrotic myofibers can be defined as round-shaped, pale, and/or hyalinized fibers combined with loss of sarcolemmal integrity/coarse appearance and were assessed on combined dystrophin 2 immunohistochemical staining and eosin (Allenbach, Arouche-Delaperche et al., 2018). CD56-positive myofibers were defined as myofiber regeneration. The perifascicular area was in the muscle myofiber domain at the periphery of the muscle fascicle (two penultimate layers) (Wedderburn et al., 2007). Fiji software was used for slice analysis.

Measured data were shown as the median and the interquartile range. Categorical variables were expressed as frequencies and percentages. Data normality of distribution was verified by Shapiro–Wilk test, Anderson–Darling test, D’Agostino and Pearson’s test, and Kolmogorov–Smirnov test. A Kruskal–Wallis H test followed by a *post-hoc* Dunn’s test or one-way analysis of variance followed by Bonferroni correction was conducted to identify whether a statistically significant difference existed when three groups were compared. Spearman’s correlation was used to analyze all the correlations. If a full data set was incomplete, the number of subjects analyzed is specified. Statistical analysis was performed using GraphPad Prism 8.01 software (GraphPad Software, Inc., La Jolla, CA, United States). Values of *p* < 0.05 were considered significant.

## Results

### Clinical Data and Pathological Findings

Clinical characteristics and muscle biopsy findings of subjects are shown in Table 1. Thirty-seven patients diagnosed with IMNM were enrolled, including 20 patients with anti-SRP antibodies, five patients with anti-HMGCR antibodies, and 12 seronegative patients. Twenty patients with a diagnosis of DM were selected, including anti-MDA5 (*n* = 7), anti-Mi-2 (*n* = 4), anti-SAE1 (*n* = 4), anti-NXP2 (*n* = 3), and anti-TIF-γ (*n* = 2). Six patients with ASS were included, including positive for anti-Jo-1 (*n* = 5) and anti-EJ antibodies (*n* = 1).

**TABLE 1:** Clinical features and pathological findings of enrolled subjects.

	Controls	IMNM	DM	ASS
Number	10	37	20	6
Age (years)^a^	31 (15–42)	48 (35–54)	45 (40–55)	56 (52–60)
Female	2 (20%)	27 (73%)	10 (50%)	3 (50%)
Disease duration (months)^a^	2 (1–9)	5 (2–10)	4 (2–11)	8 (3–12)
MMT8^a^	80 (80–80)	73 (68–75)	73 (68–78)	78 (72–80)
MSA positivity^a^	0	25 (68%)	20 (100%)	6 (100%)
MAA positivity^a^	2 (20%)	12 (32%)	6 (30%)	3 (50%)
CK (U/L)^a^	67 (57–81)	2,905 (1,729–5,331)	351 (153–1,627)	3,377 (2,310–7,528)
LDH (U/L)^a^	170 (146–204)	711 (393–839)	330 (221–631)	496 (383–1,225)
Pathological findings				
Scatter necrosis	0	36 (97%)	12 (60%)	3 (50%)
Scatter regeneration	0	32 (87%)	12 (60%)	3 (50%)
Myofiber atrophy in perifascicular regions	0	0	9 (45%)	2 (33%)
Myofiber necrosis in perifascicular regions	0	0	6 (30%)	2 (33%)
Myofiber regeneration in perifascicular regions	0	0	8 (40%)	2 (33%)

a Note: At the time of muscle biopsy. ASS, anti-synthetase syndrome; CK, creatine kinase; DM, dermatomyositis; IMNM, immune mediated necrotizing myopathy; LDH, lactate dehydrogenase; MAA, myositis-associated autoantibodies; MMT8, manual muscle test 8 (0–80); MSA, myositis specific autoantibodies.

Compared with DM and ASS, IMNM patients were more likely to be female (73% vs. 53% and 50%). The levels of serum CK and LDH tended to be higher in IMNM and ASS compared with DM (CK: 2,905 (108–17,100) in IMNM, 3,377 (498–12,546) in ASS vs. 372 (32–7,063) in DM; LDH: 711 (140–2,712) in IMNM, 496 (255–1,867) in ASS vs. 347 (144–1,370) in DM).

Patients with IMNM were pathologically characterized by scatter myofiber necrosis (97%) and myofiber regeneration (87%). Of the 20 patients with DM, 45% showed perifascicular atrophy and 30% displayed perifascicular necrosis. Two patients with ASS exhibited perifascicular necrosis and regeneration. The pathological findings of one patient with anti-SAE1 and anti-EJ antibodies predominantly displayed perifascicular atrophy but not perifascicular necrosis. This patient was classified into the DM group.

### The Expression of Endoplasmic Reticulum Stress-Induced-Autophagy-Related Protein and Gene Levels are Elevated in Muscle Samples From Patients With Idiopathic Inflammatory Myopathies

**FIGURE 1:**
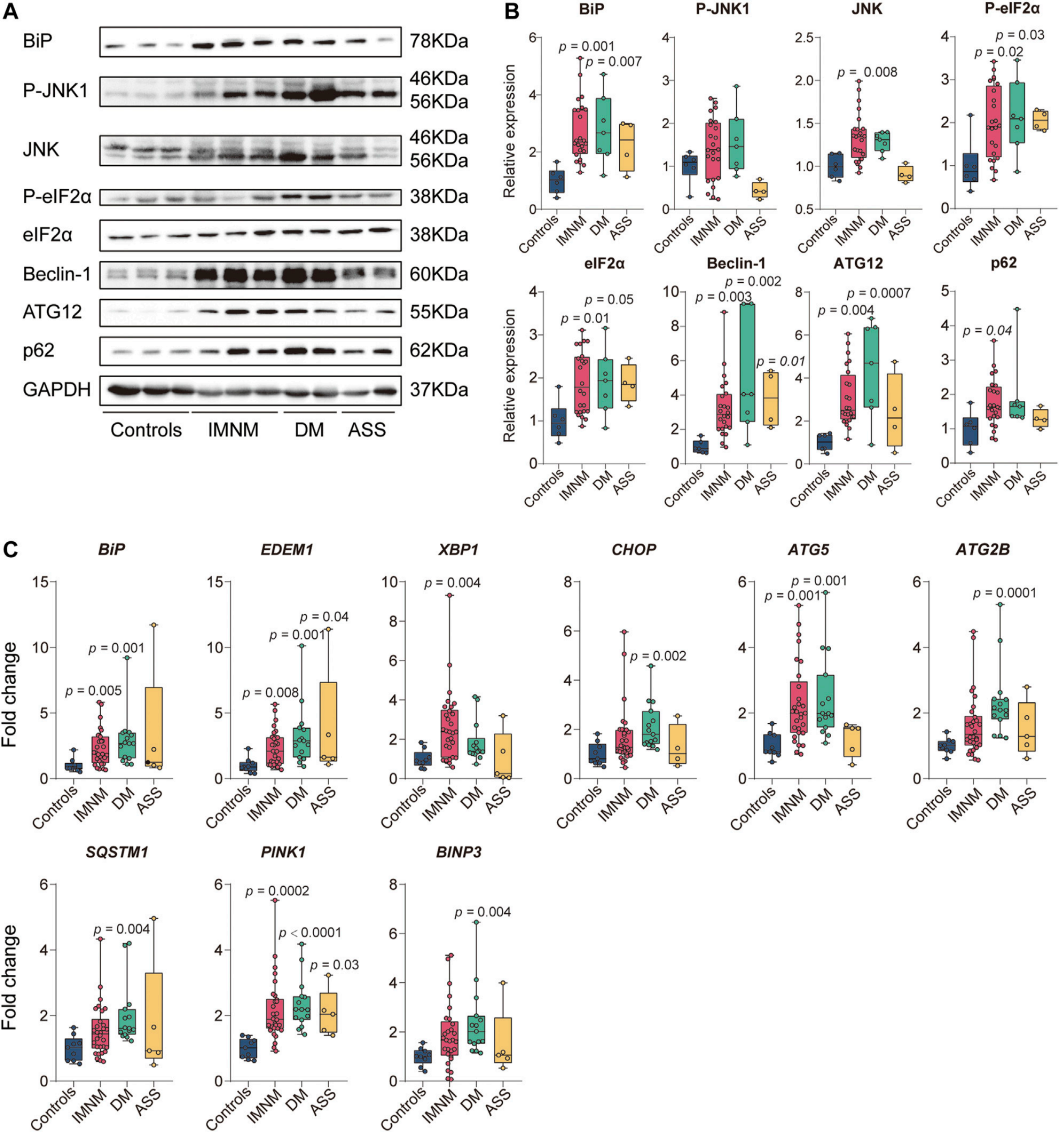
The expression level of proteins and genes involved in endoplasmic reticulum (ER) stress and autophagy pathways in controls and idiopathic inflammatory myopathies (IIM). **(A)** Representative Western blots of proteins involved in ER stress-induced autophagy signaling, including BiP, P-JNK1, JNK, P-eIF2α, eIF2α, Beclin-1, ATG12, and sequestosome 1 (p62), in controls, immune-mediated necrotizing myopathy (IMNM),dermatomyositis (DM), and anti-synthetase syndrome (ASS). Glyceraldehyde-3-phosphate dehydrogenase (GAPDH) was used for internal reference. **(B)** Quantitative analysis of relative expression levels of ER stress-induced autophagy proteins, including BiP, P-JNK1, JNK, P-eIF2α, eIF2α, Beclin-1, ATG12, and p62, in controls (*n* = 6), IMNM (*n* = 24), DM (*n* = 7), and ASS (*n* = 4). *p*-values versus controls group. **(C)** mRNA expression of ER stress-related genes, including BiP, ER degradation enhancing alpha-mannosidase-like protein 1 (EDEM1), X-box-binding protein (XBP1), and CHOP in muscle tissue from controls (*n* = 9), patients with IMNM (*n* = 29–30), DM (*n* = 14–15), and ASS (*n* = 4–5). The expression level of autophagy-related genes, including autophagy-related 5 (ATG5), autophagy-related 2B (ATG2B), sequestosome 1 (SQSTM1), phosphatase and tensin homolog (PTEN)-induced putative kinase 1 (PINK1), and BCL2-interacting protein 3 (BINP3) in controls (*n* = 9), IMNM (*n* = 29–30), DM (*n* = 14–15), and ASS (*n* = 4–5) patients. *p*-values versus controls group.

A previous study indicated that chaperone-assisted selective autophagy was involved in IMNM (Fischer et al., 2019). ER stress acts as a close linker to mechanistically induce autophagy (Song et al., 2017). We then explored the expression of proteins involved in ER-stress induced autophagy pathway in IIM biopsy skeletal muscle. ER chaperone protein GRP78/BiP triggers UPR to accelerate the accumulated protein folding activity and to prevent protein aggregation. We found that the levels of BiP protein assayed by Western blot were significantly elevated in IMNM (2.7-fold) and DM (2.8-fold) patient biopsy samples compared with control samples. A trend of an increase of BiP expression in ASS patient biopsy samples was found. There are three sensors of classic UPR signaling channels activated by ER stress, including PERK, IRE1, and ATF6 (Song et al., 2017). JNK and eIF2α, two key molecules of classic UPR signaling pathways, were examined by Western blot. The relative expression level of JNK protein was dramatically upregulated in IMNM when compared with controls (*p* = 0.008), while the P-JNK1 level was not significantly increased. There was no statistical difference in the expression of JNK and P-JNK1 between DM or ASS and the controls. The expression of eIF2α was increased in biopsied specimen from IMNM patients (*p* = 0.01), while no significant difference was found between DM or ASS and the control group. There was an upregulation of P-eIF2α protein in IMNM and DM when compared with controls (*p* = 0.02; *p* = 0.03); however, no statistical difference was observed between ASS and the control group. Beclin-1, a protein required for initiation of autophagosome formation, was increased in IMNM, DM, and ASS patient biopsy samples (*p* = 0.003, *p* = 0.002, and *p* = 0.01, respectively). ATG12, a protein required for autophagy formation, was significantly upregulated in IMNM and DM (*p* = 0.004; *p* = 0.0007). p62, a protein required for the formation of protein aggregates that are eliminated by autophagy, was dramatically increased in IMNM patients (*p* = 0.04) (Figures 1A,B).

Then the genes involved in ER stress-induced autophagy were examined by RT-qPCR. Consistent with the results of Western blot, *BiP* gene expression was significantly increased in IMNM and DM compared with controls (*p* = 0.005; *p* = 0.001). There was no significant difference in the *BiP* level between controls and ASS. ER stress response gene *EDEM1*, encoding the main members of ER-association degradation activated during ER stress, was found at significantly higher levels in biopsies from patients with IMNM, DM, and ASS compared with controls (*p* = 0.008, *p* = 0.001, and *p* = 0.04, respectively). XBP1 is involved in ER-association degradation by the induction of EDEM. *XBP1* gene expression showed significantly increased levels in specimens from IMNM (*p* = 0.004), while no significant difference was observed between controls and DM or controls and ASS. *CHOP*, a mediator of ER stress-induced apoptosis, was significantly elevated in DM (*p* = 0.002). There was no significant increase in the expression of *CHOP* in IMNM or ASS compared with controls, which was consistent with previous study (Fischer et al., 2019). Compared with controls, skeletal muscle biopsies from patients with IMNM or DM showed significant increased levels of *ATG5* gene expression (*p* = 0.001 and *p* = 0.001, respectively), encoding for ATG5, an essential molecule required for autophagosome formation, while there was no significant difference between controls and ASS. *ATG2B* as well encodes a protein required for autophagosome formation, which was elevated in DM but not in IMNM or ASS when compared with controls (*p* = 0.0001). *SQSTM1* encoding for p62, a protein that connects polyubiquitinylated proteins with LC3, was not differentially regulated in specimens from IMNM or ASS, but significantly upregulated in DM when compared with controls (*p* = 0.004). Mitophagy is a process for autophagic elimination of damaged mitochondria. *PINK1* and *BINP3* encode for proteins involved in mitophagy. The gene level of *PINK1* was significantly increased in the IMNM, DM, and ASS groups (*p* = 0.0002, *p* < 0.0001, and *p* = 0.03, respectively). *BINP3* expression was upregulated in the DM groups (*p* = 0.004). However, there was no significant increase in *BINP3* expression in IMNM patients, as well as in either ASS group (Figure 1C).

**FIGURE 2:**
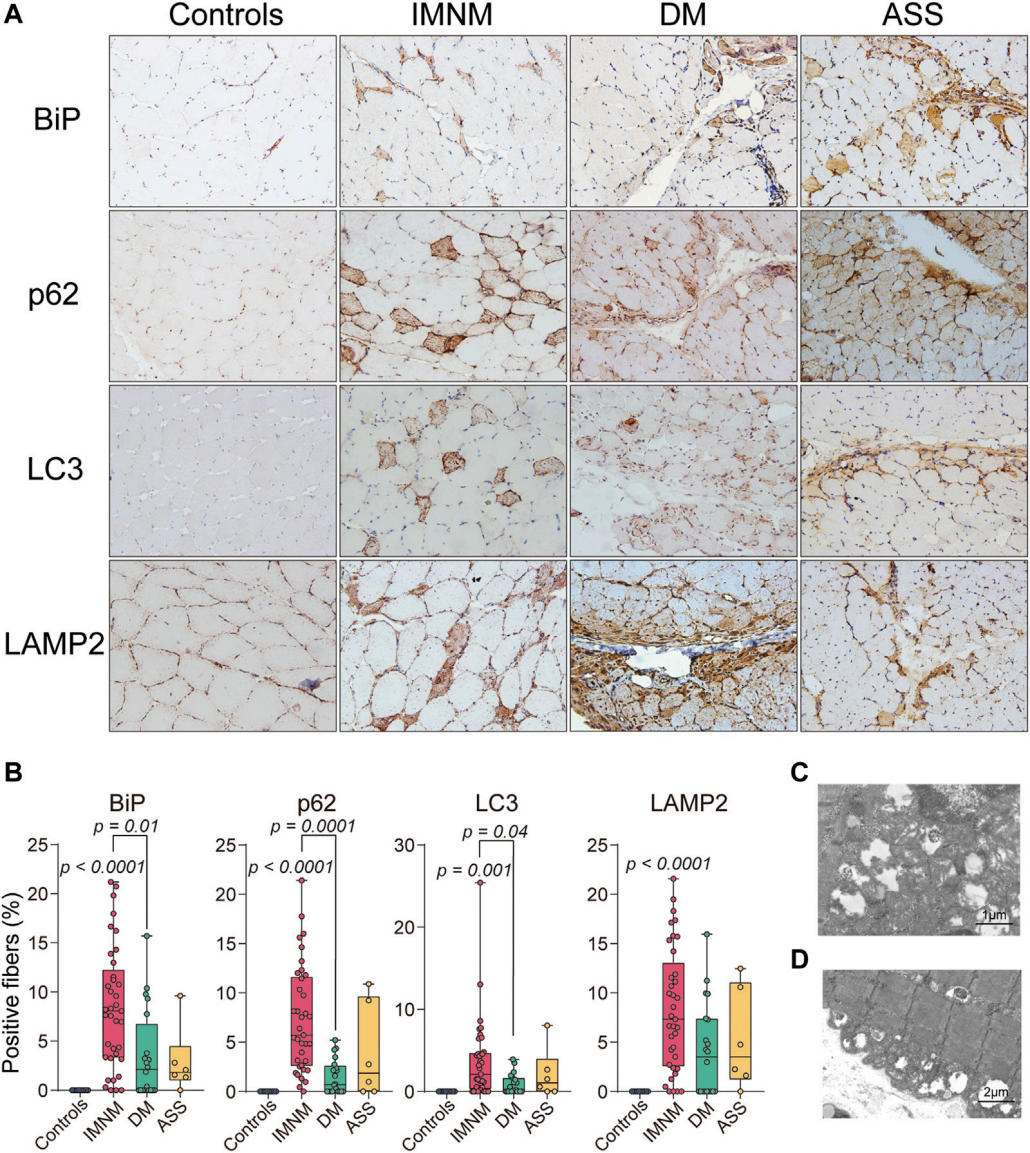
Staining pattern of BiP, p62, LC3, and lysosomal-associated membrane protein 2 (LAMP2) in controls and patients with IIM. **(A)** Muscle tissue from a control subject demonstrating no positive staining and scattered positive staining of BiP, p62, LC3, and LAMP2 in biopsy muscle from a patient with IMNM. The distribution of positive staining of these molecules from a patient with DM and a patient with ASS mainly in perifascicular areas. Magnification: ×200. **(B)** Quantification of the percentage of positive staining of BiP, p62, LC3, and LAMP2 in controls, IMNM, DM, and ASS. *p* values versus controls group or comparisons between IMNM and DM. **(C,D)** Transmission electron microscopy (TEM) images showing lysosomes containing debris and nonspecific granular material in diffuse distribution.

BiP sarcoplasmic immunostaining was comparatively strong in IIM patients, and no positive sarcoplasmic staining of BiP was observed in skeletal muscle from controls (Figure 2A). The staining pattern of BiP in biopsied muscles from IMNM was diffused or scattered (Figure 2A), while the positive staining of BiP was mainly distributed in perifascicular areas in DM and ASS with perifascicular injury (Figure 2A). Consistent with previous study (Fischer et al., 2019; Girolamo et al., 2019), a diffusely fine and homogenous sarcoplasmic staining of p62, LC3 (antibody recognizes both LC3I and -II), and LAMP2 was observed in IMNM. The distribution of punctate p62, LC3, and LAMP2 in biopsied skeletal muscle from DM and ASS with perifascicular injury was predominantly confined in perifascicular areas. Specimens from controls consistently showed no positive staining of their sarcoplasm (Figure 2A).

The percentage of BiP-positive myofibers was significantly increased in skeletal muscle of IMNM (*p* < 0.0001), while no statistically elevated levels of BiP was found between controls and DM or ASS. The percentage of p62 and LC3-positive myofibers were significantly elevated in IMNM (*p* < 0.0001; *p* = 0.001), whereas no significant increase was detected in DM compared with controls (Figure 2B). The elevated percentage of LAMP2 positive myofibers was found in patients IMNM (*p* < 0.0001) (Figure 2B). Furthermore, compared with those with DM, patients with IMNM exhibited significantly elevated levels of sarcoplasmic staining of BiP, p62, and LC3 (*p* = 0.01, *p* = 0.0001, and *p* = 0.04, respectively) (Figure 2B).

Vacuoles containing debris and nonspecific granular material in lysosomes were detected by TEM in muscle tissues of IIM (Figures 2C,D).

### Sarcoplasmic BiP Expression Correlates With Multifactorial Processes in Idiopathic Inflammatory Myopathies

**FIGURE 3:**
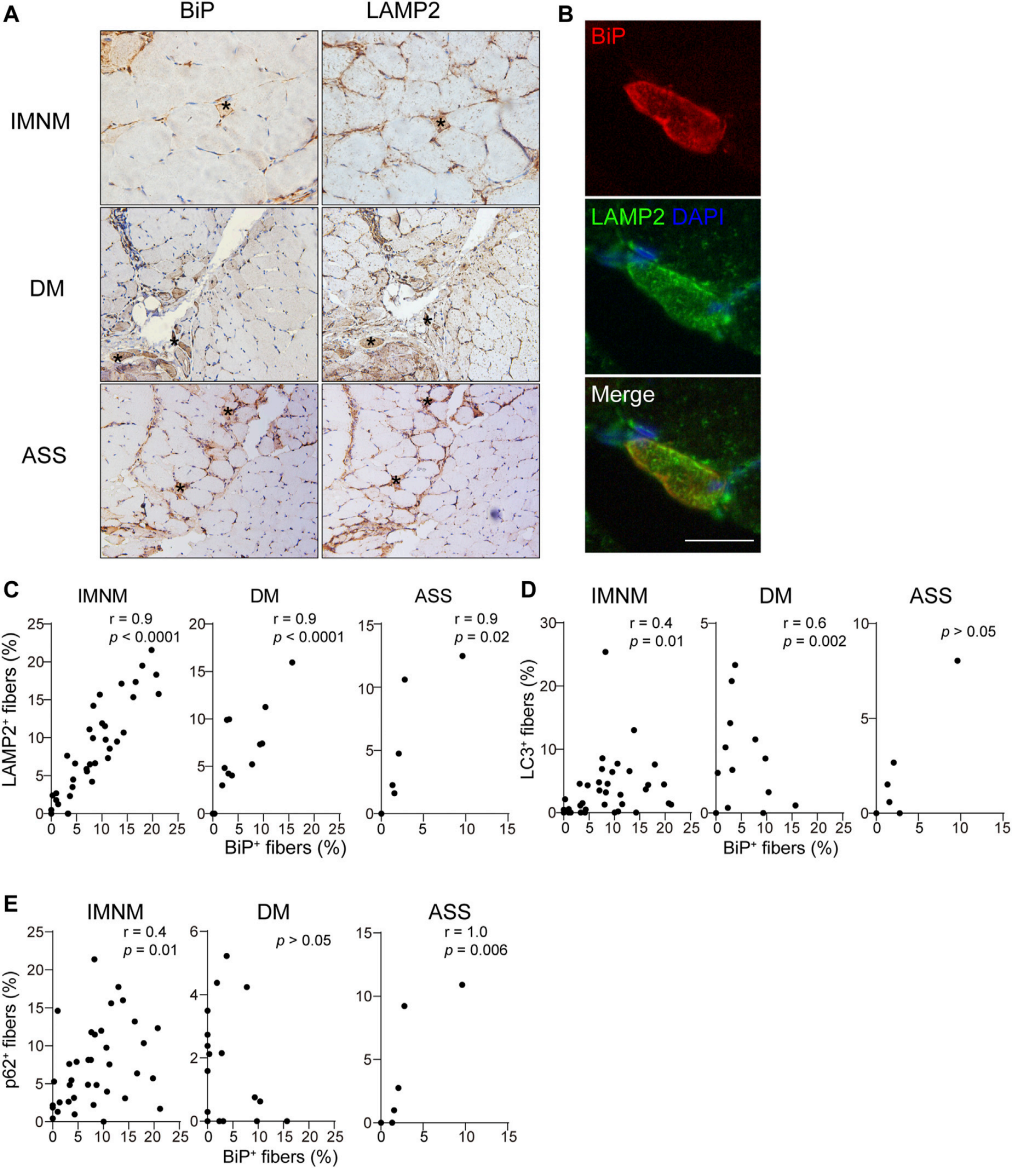
The expression of BiP correlated with dysregulation of autolysosomes. **(A)** Successive sections in a patient with IMNM, a patient with DM, and a patient with ASS demonstrating some BiP-positive myofiber positively for LAMP2 staining (examples*). Magnification: ×200. **(B)** Double IF staining of a section obtained from a patient with IMNM showing the staining of BiP and LAMP2 in the same myofibers. Scale bar, 50 μm. **(C)** Relationship between the percentage of positive LAMP2 and sarcoplasmic BiP expression in patients with IMNM, DM, and ASS. **(D)** Associations between the percentage of positive BiP and LC3 expression in muscle tissue obtained from patients with IMNM, DM, and ASS. **(E)** Correlations between the expression levels of BiP and p62 in IMNM, DM, and ASS skeletal muscle sections.

The histologic slices showed some LAMP2-positive myofibers co-distributed within individual BiP-positive muscle fibers (Figures 3A,B). The percentages of sarcoplasmic BiP expression strongly correlated with the percentages of lysosome marker LAMP2-positive myofibers in IMNM (*r* = 0.9, *p* < 0.0001), DM (*r* = 0.9, *p* < 0.0001), and ASS (*r* = 0.9, *p* = 0.02) (Figure 3C). The BiP expression also was closely associated with p62 and LC3 expression in IMNM (*r* = 0.4, *p* = 0.01; *r* = 0.4, *p* = 0.01) (Figures 3D,E). A significant correlation between the percentage of BiP and LC3 expression in DM biopsied muscle was observed (*r* = 0.6, *p* = 0.002), while there was no association between BiP and p62 levels in DM (Figures 3D,E). In ASS, there was a strong association between BiP expression and p62 levels (*r* = 1.0, *p* = 0.006), whereas no correlation between BiP and LC3 expression was found (Figures 3D,E). These data suggest that ER stress may be an important trigger for the accumulation and dysregulation of autolysosomes in IIM.

**FIGURE 4:**
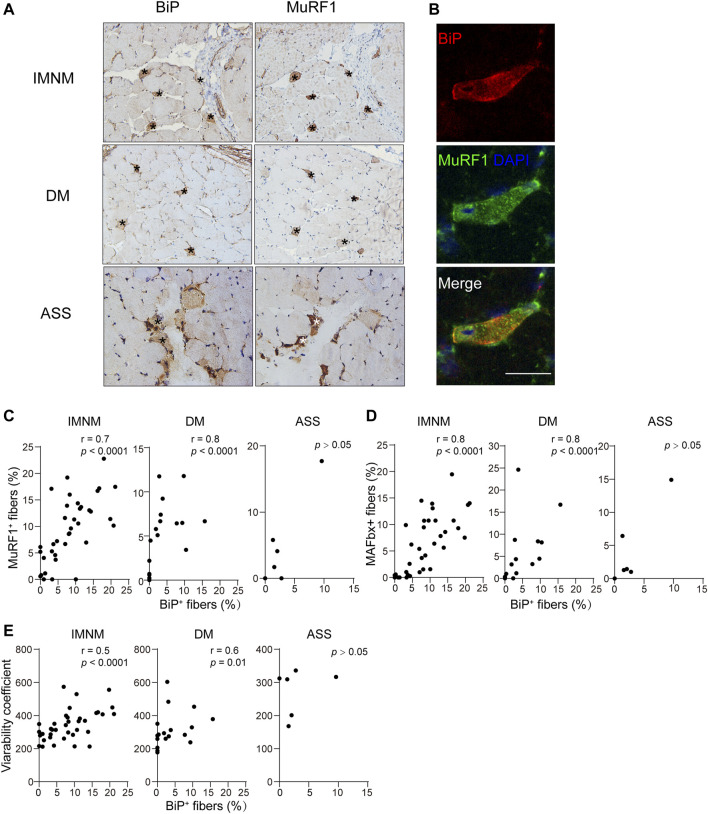
BiP expression correlated with myofiber atrophy. **(A)** BiP staining of muscle from a patient with IMNM, a patient with DM, and, a patient with ASS showing abnormal positivity in these cells (*). These cells were positive for muscle RING finger protein-1 (MuRF1). Magnification: ×200. **(B)** Double IF staining demonstrating the staining of BiP and MuRF1 in the same myofibers in a section from a patient with IMNM. Scale bar, 50 μm. **(C)** Associations between the percentage of positive BiP and sarcoplasmic MuRF1 expression in muscle tissue obtained from patients with IMNM, DM, and ASS. **(D)** Relationships between the expression levels of BiP and sarcoplasmic muscle atrophy F-box (MAFbx) expression levels in patients with IMNM, DM, and ASS biopsied muscle. **(E)** Correlations between the expression levels of BiP and variability coefficient in IMNM, DM, and ASS skeletal muscle sections.

Skeletal muscle atrophy or wasting, presenting as overall loss of muscle mass, is induced by the fact that rate of protein degradation exceeds that of protein synthesis. Markers of atrophy, MuRF1/TRIM63 or MAFbx/ATROGIN-1, were upregulated during skeletal muscle atrophy (Bodine et al., 2001; Gomes et al., 2001; Bodine and Baehr, 2014). The staining pattern of MuRF1 and MAFbx was scattered in IMNM but mainly distributed in perifascicular areas in DM and ASS with perifascicular injury (Supplementary Figure S1A). The expression of MuRF1 was statistically increased in biopsied muscle tissues of IMNM (*p* < 0.0001) and DM (*p* = 0.04), whereas no significant difference was found between ASS and controls (Supplementary Figure S1B). Similarly, the percentage of MAFbx-positive myofibers was significantly elevated in IMNM (*p* < 0.0001) and DM (*p* = 0.03) (Supplementary Figure S1B). The histologic slices showed many MuRF1-positive myofibers co-distributed within BiP-positive muscle fibers (Figures 4A,B). IMNM and DM biopsy muscles exhibited that BiP expression was strongly associated with MuRF1 expression (*r* = 0.7, *p* < 0.0001; *r* = 0.8, *p* < 0.0001). Consistently, the percentage of BiP expression was dramatically related with the MAFbx level in IMNM (*r* = 0.8, *p* < 0.0001) and DM (*r* = 0.8, *p* < 0.0001) (Figure 4D). There was no significant correlation between the expression of BiP and MuRF1, or BiP and MAFbx in ASS (Figure 4C). Coefficients of variability were considered as an index for the degree of atrophy in skeletal muscle diseases (Briguet et al., 2004; Arouche-Delaperche et al., 2017). Variability coefficient was strongly associated with the expression level of BiP in IMNM (*r* = 0.5, *p* < 0.0001), while there was no significant relationship in ASS (Figure 4E). A correlation between variability coefficient and BiP expression was also observed in DM (*r* = 0.6, *p* = 0.01). The results indicate that ER stress may play a vital role in skeletal muscle atrophy in IIM, especially in IMNM and DM.

**FIGURE 5:**
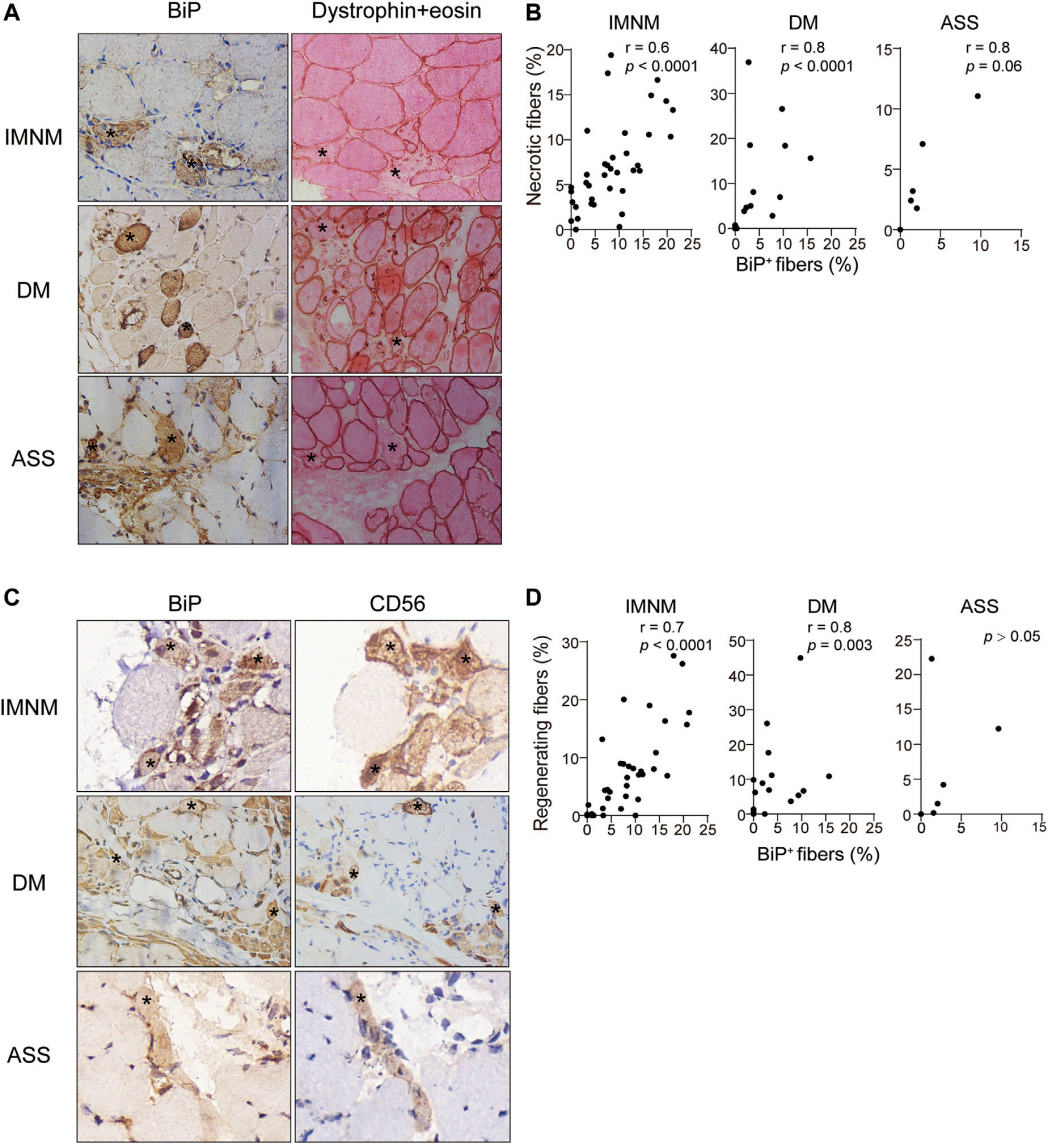
BiP expression correlated with myofiber necrosis and regeneration. **(A)** Dystrophin combined eosin staining of sections obtained from a patient with IMNM, a patient with DM, and a patient with ASS demonstrating numerous necrotic fibers (examples *). These fibers exhibited BiP staining. Magnification: ×400. **(B)** Associations between the percentages of necrotic myofibers and BiP-positive myofibers in IMNM, DM, and ASS subjects. **(C)** NCAM-positive myofibers from a patient with IMNM, a patient with DM, and a patient with ASS showing positively staining for BiP (examples *). Magnification: (IMNM, ASS) ×400; (DM) x200. **(D)** Correlations between the expression levels of BiP and the percentages of regenerating myofibers in muscle tissue obtained from IMNM, DM, and ASS patients.

Necrosis was a pivotal pathologic feature in IIM, particularly in IMNM. The distribution of necrotic myofibers in IMNM was scattered but primarily in perifascicular areas in DM and ASS with perifascicular injury (Supplementary Figure S2A). Numerous necrotic cells exhibited strong positive staining for BiP in IMNM, DM, and ASS (Figure 5A). Statistically, the BiP expression closely correlated with the percentage of necrotic cells in IMNM (*r* = 0.6, *p* < 0.0001) and DM (*r* = 0.8, *p* < 0.0001). There is a tendency of weak association between the proportion of myonecrosis and BiP expression in ASS (*r* = 0.8, *p* = 0.06) (Figure 5B).

Regeneration was important for skeletal muscle restoration in skeletal muscle disease and then was investigated. Consistent with the staining pattern of necrotic myofibers, a diffused distribution of regenerating myofibers was noted in IMNM, while in DM and ASS with perifascicular injury, regenerating myofibers predominantly distributed in the perifascicular area (Supplementary Figure S2B). Some regenerating myofibers biopsied muscle from IMNM and DM were stained positively for BiP (Figure 5C). The expression of BiP was significantly associated with the percentage of CD56 positive fibers as regenerating fibers in IMNM (*r* = 0.7, *p* < 0.0001) and DM (*r* = 0.8, *p* = 0.003) (Figure 5D). Though a few regenerating myofibers in sections from ASS were positive for BiP (Figure 5C), no significant relationship between them in ASS was found (Figure 5D). Only occasionally was BiP positivity noted in relatively appearing normal myofibers in IIM muscle biopsy. It is concluded that ER stress may participate in the pathological process of myonecrosis and myofiber regeneration in IMNM and DM.

### The Expression of BiP Correlates With Muscle Weakness in Immune-Mediated Necrotizing Myopathy

**FIGURE 6:**
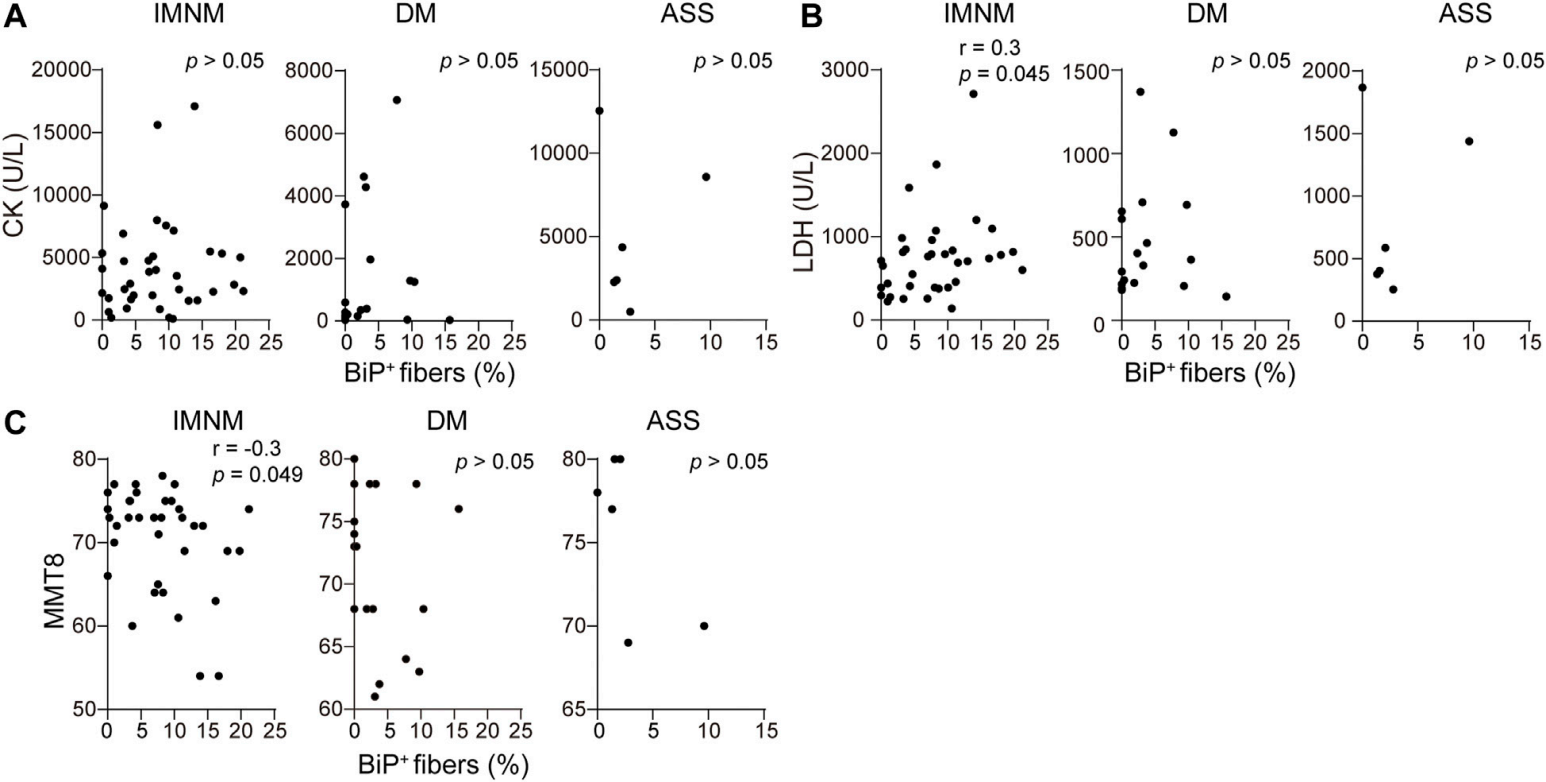
BiP expression was related with muscle weakness in IMNM. **(A)** Associations between the percentage of positive BiP staining in muscle tissue and serum creatine phosphokinase (CK) at the time of biopsy in IMNM (*n* = 36), DM (*n* = 20), and ASS (*n* = 6) patients. **(B)** Relationships between BiP expression in muscle sections and serum lactic dehydrogenase (LDH) at the time of biopsy in IMNM (*n* = 36), DM (*n* = 20), and ASS (*n* = 6) patients. **(C)** Correlations between the expression level of BiP and manual muscle testing (MMT-8) scores at the time of biopsy in IMNM (*n* = 36), DM (*n* = 20), and ASS (*n* = 6) patients.

There was a weakly positive correlation between serum LDH levels and expression of BiP in skeletal muscle patients with IMNM (*r* = 0.3, *p* = 0.045) (Figure 6B). Consistently, BiP expression negatively correlated with MMT-8 assessments at the time of muscle biopsy in IMNM (*r* = −0.3, *p* = 0.049) (Figure 6C). No association was found between serum CK levels and the percentage of BiP-positive myofibers in IMNM (Figure 6A). There were no significant correlations between levels of serum CK and LDH, or MMT-8 scores and BiP expression in DM or ASS (Figures 6A–C).

## Discussion

It is well established that autoimmune and inflammation response, to a great extent, contribute to the pathogenesis of IIM. Current research for the pathophysiology of IIM has mainly focused on immune and inflammatory response, such as autoantibodies (Benveniste et al., 2016; Arouche-Delaperche et al., 2017; Darnoiseaux et al., 2019), complement (Allenbach, Arouche-Delaperche et al., 2018; Bergua et al., 2019), cytokines (Day et al., 2020), inflammatory cells (Preuße et al., 2012; Rinnenthal et al., 2014; Knauss et al., 2019; Jiang et al., 2020; Seto et al., 2020), interferon-signature (Greenberg et al., 2005; Gallay et al., 2019), and the upregulation of major histocompatibility complex—class I on myofibers (Bhattarai et al., 2016; Wang et al., 2018). Although a previous study revealed evidence for activation of the ER stress response in polymyositis and DM (Nagaraju et al., 2005; Alger et al., 2011), the functional role of ER stress was still poorly understood. The present study suggests that ER stress-mediated activation of autophagy is extremely likely involved in IIM. We also find that ER stress is closely associated with multiple physiological and pathological processes ranging from destruction to restoration in IIM biopsy muscle. Furthermore, ER stress correlates with muscle weakness in IMNM. The dynamic physiological processes and distinct pathogenesis mechanisms responsible for different IIM subtypes may explain the varying degrees of BiP expression we detected across these disease conditions.

A previous study indicated that activation of ER stress and unfolded protein response pathways may induce the impaired autophagy in sIBM (Nogalska et al., 2010). The involvement of chaperone-assisted selective autophagy has been described in IMNM (Giannini et al., 2018; Fischer et al., 2019). Our results further validate that ER stress mediated-activation of autophagy may be involved in IMNM. Both of SRP and HMGCR proteins are located at the ER. The mechanism on how these circulatory antibodies bind to their intracellular targets remains unclear. In specific disease condition, the intracellular molecules can appear at the cell membrane. It was described that the calnexin, a molecular chaperone in the ER, was expressed on the cell surface in various cells, such as mastocytoma cells, murine splenocytes, fibroblast cells, and human HeLa cells (Okazaki et al., 2000). Presumably, the antigen–antibody complexes in seropositive IMNM may target the protein translational structures as a potential direct stressor of the ER to induce autophagy. In addition, ER stress response may also play a role in the induction of autophagy in DM and ASS, although the expression levels of BiP and autophagolysosomal markers, LC3, p62, and LAMP2, was lower in DM and ASS than in IMNM. In DM and ASS with perifascicular injury, these molecules are predominantly expressed in perifascicular areas. Such phenomenon is consistent with the key pathologic feature of DM and ASS (Mescam-Mancini et al., 2015; Yasin et al., 2018). A previous study indicated that perifascicular atrophy was prominently related to the activation of type-1 interferon-associated pathway in DM (Preusse et al., 2016). It is speculated that ER stress-induced autophagy in DM may be associated with the upregulation of type 1 interferon-inducible genes. The hypoxia-related mechanism may be responsible for ER stress in ASS, given that perifascicular necrosis probably attributed to vasculopathy in ASS (Mescam-Mancini et al., 2015), and the anti-synthetase antibodies may contribute to autophagy by blocking the transfer RNA syntheses. These assumptions obviously warrant more evidence.

While it seems paradoxical that ER stress could be associated with multiple processes, there is increasing evidence for complex functional properties of ER stress in skeletal muscle remodeling (Afroze and Kumar, 2019). Overall, our results imply a deleterious role for ER stress in IMNM, as sarcoplasmic expression correlated with myonecrosis, myofiber atrophy, and clinical disease activity. This is consistent with evidence demonstrating that activation of ER stress and the UPR pathways results in skeletal muscle atrophy in numerous conditions (Bohnert et al., 2018). A previous study indicated that the pathogenic role of anti-SRP and anti-HMGCR antibodies resulted in fiber atrophy and impairment of muscle regeneration (Arouche-Delaperche et al., 2017). Therefore, it is assumed that anti-SRP and anti-HMGCR antibodies, displayed as triggers, lead to myofiber atrophy by ER stress-induced autophagy in seropositive IMNM.

The accumulation of ER stress in myofibers co-distributed with the overexpression of major histocompatibility complex—class I in IIM, which can trigger ER stress in a mouse model of myositis (Nagaraju et al., 2005). The positive correlation between BiP expression and myonecrosis in our study may be related to the upregulation of major histocompatibility complex—class I on sarcolemma. Moreover, ER stress could increase expression of tumor necrosis factor and other inflammatory cytokines under certain stimulations (Stengel et al., 2020). It is conceivable that these cytokines could provoke perpetuate muscle damage cytolysis, and vice versa, these cytokines released from necrotic cells will itself provoke ER stress. Of note, CHOP, a mediator of ER stress-induced apoptosis, the gene and protein levels of CHOP were not upregulated, and an apoptosis-related molecule cleaved-caspase 3 protein was either not increased in IMNM muscle biopsy (data not shown), suggesting that ER stress-induced apoptosis may not be a role in the pathogenesis of IMNM.

On the other hand, our data demonstrated links of ER stress with beneficial processes, such as myofiber regeneration. A previous study showed that mild ER stress inhibited caspase activation and apoptosis by promoting autophagy in neurodegenerative diseases (Fouillet et al., 2012). In adult skeletal muscle tissue, low levels of ER stress may be beneficial in the maintenance of the pool of satellite cells for regenerative myogenesis (Bohnert et al., 2018). Thus, it is interpretable to observe a significant association between BiP and CD56 expression in skeletal muscle of IMNM and DM. Further research evaluating the multiple roles of ER stress in regulating disease progression of IIM and the exact role of regenerative myofibers are clearly required before therapeutic intervention targeting ER stress pathways ought to be considered.

This study has several limitations. First, our study is observational and descriptive in nature, and the complex mechanisms underpinning the correlations cannot be determined. Second, considering the relatively small-sized sample in a single clinical center, our data, however, need to be further confirmed on a larger number of subjects from multiple clinical centers.

In conclusion, this study evaluated BiP expression in IIM subgroups, suggesting that ER stress may exert different effects depending on the type of stress condition. These important findings may facilitate processes and mechanisms regarding the role of ER stress in autoimmune muscle diseases.

## Data Availability

The original contributions presented in the study are included in the article/Supplementary Material, further inquiries can be directed to the corresponding author.

## Supplementary Material

Supplementary Figure S1 The staining pattern of MuRF1 and MAFbx in controls and patients with IIM. **(A)** Muscle tissue from a control individual showing no positive staining of MuRF1 and MAFbx. Diffuse positive staining of MuRF1 and MAFbx in biopsy muscle from a patient with IMNM. A predominantly positive staining of these molecules from a patient DM and a patient with ASS in perifascicular areas. Magnification: × 200. **(B)** Quantification of the percentage of positive staining of MuRF1 and MAFbx in controls, IMNM, DM, and ASS. *p* values versus controls group.

Supplementary Figure S2 The distribution of myonecrosis and myoregeneration in IIM. **(A)** Necrotic myofibers was scattered in muscle tissue from a patient with IMNM by dystrophin combined eosin staining. Myonecrosis was mainly in perifascicular areas in muscle tissue from a patient with DM and a patient with ASS. * Necrotic fibers. Magnification: × 200. **(B)** Scattered staining of CD56 in muscle tissue from a patient with IMNM. Regenerative myofibers were primarily distributed in perifascicular areas in sections from a patient with DM and a patient with ASS. Magnification: × 200.

## References

[B1] Afroze D. Kumar A. (2019). ER Stress in Skeletal Muscle Remodeling and Myopathies. FEBS J. 286 (2), 379–398. 10.1111/febs.14358 29239106 PMC6002870

[B2] Alger H. M. Rayavarapu S. Nagaraju K. (2011). Measurement of Activation of the Endoplasmic Reticulum Stress Response in Autoimmune Myositis. Methods Enzymol. 489, 207–225. 10.1016/b978-0-12-385116-1.00012-1 21266232

[B3] Allenbach Y. Arouche-Delaperche L. Preusse C. Radbruch H. Butler-Browne G. Champtiaux N. (2018). Necrosis in Anti-SRP+ and Anti-HMGCR+myopathies. Neurology 90 (6), e507–e517. 10.1212/wnl.0000000000004923 29330311

[B4] Allenbach Y. Mammen A. L. Benveniste O. Stenzel W. Allenbach Y. Amato A. (2018). 224th ENMC International Workshop: Clinico-Sero-Pathological Classification of Immune-Mediated Necrotizing Myopathies Zandvoort, The Netherlands, 14-16 October 2016. Neuromuscul. Disord. 28 (1), 87–99. 10.1016/j.nmd.2017.09.016 29221629

[B5] Arouche-Delaperche L. Allenbach Y. Amelin D. Preusse C. Mouly V. Mauhin W. (2017). Pathogenic Role of Anti-signal Recognition Protein and Anti-3-hydroxy-3-methylglutaryl-CoA Reductase Antibodies in Necrotizing Myopathies: Myofiber Atrophy and Impairment of Muscle Regeneration in Necrotizing Autoimmune Myopathies. Ann. Neurol. 81 (4), 538–548. 10.1002/ana.24902 28224701

[B6] Benveniste O. Stenzel W. Allenbach Y. (2016). Advances in Serological Diagnostics of Inflammatory Myopathies. Curr. Opin. Neurol. 29 (5), 662–673. 10.1097/wco.0000000000000376 27538058

[B7] Bergua C. Chiavelli H. Allenbach Y. Arouche-Delaperche L. Arnoult C. Bourdenet G. (2019). *In Vivo* pathogenicity of IgG from Patients with Anti-SRP or Anti-HMGCR Autoantibodies in Immune-Mediated Necrotising Myopathy. Ann. Rheum. Dis. 78 (1), 131–139. 10.1136/annrheumdis-2018-213518 30309969

[B8] Bhattarai S. Ghannam K. Krause S. Benveniste O. Marg A. de Bruin G. (2016). The Immunoproteasomes Are Key to Regulate Myokines and MHC Class I Expression in Idiopathic Inflammatory Myopathies. J. Autoimmun. 75, 118–129. 10.1016/j.jaut.2016.08.004 27522114

[B9] Bodine S. C. Baehr L. M. (2014). Skeletal Muscle Atrophy and the E3 Ubiquitin Ligases MuRF1 and MAFbx/atrogin-1. Am. J. Physiology-Endocrinology Metab. 307 (6), E469–E484. 10.1152/ajpendo.00204.2014 PMC4166716 25096180

[B10] Bodine S. C. Latres E. Baumhueter S. Lai V. K.-M. Nunez L. Clarke B. A. (2001). Identification of Ubiquitin Ligases Required for Skeletal Muscle Atrophy. Science 294 (5547), 1704–1708. 10.1126/science.1065874 11679633

[B11] Bohnert K. R. McMillan J. D. Kumar A. (2018). Emerging Roles of ER Stress and Unfolded Protein Response Pathways in Skeletal Muscle Health and Disease. J. Cel Physiol 233 (1), 67–78. 10.1002/jcp.25852 PMC5548649 28177127

[B12] Briguet A. Courdier-Fruh I. Foster M. Meier T. Magyar J. P. (2004). Histological Parameters for the Quantitative Assessment of Muscular Dystrophy in the Mdx-Mouse. Neuromuscul. Disord. 14 (10), 675–682. 10.1016/j.nmd.2004.06.008 15351425

[B13] Cavazzana I. Fredi M. Ceribelli A. Mordenti C. Ferrari F. Carabellese N. (2016). Testing for Myositis Specific Autoantibodies: Comparison between Line Blot and Immunoprecipitation Assays in 57 Myositis Sera. J. Immunological Methods 433, 1–5. 10.1016/j.jim.2016.02.017 26906088

[B14] Darnoiseaux J. Vulsteke J.-B. Tseng C.-W. Platteel A. C. M. Piette Y. Shovman O. (2019). Autoantibodies in Idiopathic Inflammatory Myopathies: Clinical Associations and Laboratory Evaluation by Mono- and Multispecific Immunoassays. Autoimmun. Rev. 18 (3), 293–305. 10.1016/j.autrev.2018.10.004 30639643

[B15] Day J. Otto S. Cash K. Eldi P. Hissaria P. Proudman S. (2020). Aberrant Expression of High Mobility Group Box Protein 1 in the Idiopathic Inflammatory Myopathies. Front. Cel Dev. Biol. 8, 226. 10.3389/fcell.2020.00226 PMC7180187 32363191

[B16] Fischer N. Preuße C. Radke J. Pehl D. Allenbach Y. Schneider U. (2019). Sequestosome-1 (P62) Expression Reveals Chaperone-Assisted Selective Autophagy in Immune-Mediated Necrotizing Myopathies. Brain Pathol. 30 (2), 261–271. 10.1111/bpa.12772 31376301 PMC8018061

[B17] Fouillet A. Levet C. Virgone A. Robin M. Dourlen P. Rieusset J. (2012). ER Stress Inhibits Neuronal Death by Promoting Autophagy. Autophagy 8 (6), 915–926. 10.4161/auto.19716 22660271 PMC3427257

[B18] Gallay L. Mouchiroud G. Chazaud B. (2019). Interferon-signature in Idiopathic Inflammatory Myopathies. Curr. Opin. Rheumatol. 31 (6), 634–642. 10.1097/bor.0000000000000653 31464706

[B19] Giannini M. Girolamo F. Amati A. Lia A. Serlenga L. D'Abbicco D. (2018). Peculiar Expression of Autophagy Biomarkers in Necrotizing Autoimmune Myopathy Muscle. Ann. Rheum. Dis. 77, 731. 10.1136/annrheumdis-2018-eular.2530

[B20] Girolamo F. Lia A. Annese T. Giannini M. Amati A. Abbicco D. D. (2019). Autophagy Markers LC3 and P62 Accumulate in Immune-Mediated Necrotizing Myopathy. Muscle Nerve 60 (3), 315–327. 10.1002/mus.26608 31172530

[B21] Gomes M. D. Lecker S. H. Jagoe R. T. Navon A. Goldberg A. L. (2001). Atrogin-1, a Muscle-specific F-Box Protein Highly Expressed during Muscle Atrophy. Proc. Natl. Acad. Sci. 98 (25), 14440–14445. 10.1073/pnas.251541198 11717410 PMC64700

[B22] Greenberg S. A. Pinkus J. L. Pinkus G. S. Burleson T. Sanoudou D. Tawil R. (2005). Interferon-α/β-mediated Innate Immune Mechanisms in Dermatomyositis. Ann. Neurol. 57 (5), 664–678. 10.1002/ana.20464 15852401

[B23] Hoogendijk J. E. Amato A. A. Lecky B. R. Choy E. H. Lundberg I. E. Rose M. R. (2004). 119th ENMC International Workshop: Trial Design in Adult Idiopathic Inflammatory Myopathies, with the Exception of Inclusion Body Myositis, 10-12 October 2003, Naarden, The Netherlands. Neuromuscul. Disord. 14 (5), 337–345. 10.1016/j.nmd.2004.02.006 15099594

[B24] Jiang T. Huang Y. Liu H. Xu Q. Gong Y. Chen Y. (2020). Reduced miR-146a Promotes REG3A Expression and Macrophage Migration in Polymyositis and Dermatomyositis. Front. Immunol. 11, 37. 10.3389/fimmu.2020.00037 32153557 PMC7047152

[B25] Knauss S. Preusse C. Allenbach Y. Leonard-Louis S. Touat M. Fischer N. (2019). PD1 Pathway in Immune-Mediated Myopathies. Neurol. Neuroimmunol Neuroinflamm 6 (3), e558. 10.1212/nxi.0000000000000558 31044146 PMC6467687

[B26] Li S. Li W. Jiang W. He L. Peng Q. Wang G. (2021). The Efficacy of Tocilizumab in the Treatment of Patients with Refractory Immune-Mediated Necrotizing Myopathies: An Open-Label Pilot Study. Front. Pharmacol. 12, 635654. 10.3389/fphar.2021.635654 33815117 PMC8010666

[B27] Livak K. J. Schmittgen T. D. (2001). Analysis of Relative Gene Expression Data Using Real-Time Quantitative PCR and the 2−ΔΔCT Method. Methods 25 (4), 402–408. 10.1006/meth.2001.1262 11846609

[B28] Mecoli C. A. Lahouti A. H. Brodsky R. A. Mammen A. L. Christopher-Stine L. (2017). High-dose Cyclophosphamide without Stem Cell rescue in Immune-Mediated Necrotizing Myopathies. Neurol. Neuroimmunol Neuroinflamm 4 (5), e381. 10.1212/nxi.0000000000000381 28975138 PMC5619913

[B29] Mescam-Mancini L. Allenbach Y. Hervier B. Devilliers H. Mariampillay K. Dubourg O. (2015). Anti-Jo-1 Antibody-Positive Patients Show a Characteristic Necrotizing Perifascicular Myositis. Brain 138 (Pt 9), 2485–2492. 10.1093/brain/awv192 26198592

[B30] Nagaraju K. Casciola-Rosen L. Lundberg I. Rawat R. Cutting S. Thapliyal R. (2005). Activation of the Endoplasmic Reticulum Stress Response in Autoimmune Myositis: Potential Role in Muscle Fiber Damage and Dysfunction. Arthritis Rheum. 52 (6), 1824–1835. 10.1002/art.21103 15934115

[B31] Nogalska A. D'Agostino C. Terracciano C. Engel W. K. Askanas V. (2010). Impaired Autophagy in Sporadic Inclusion-Body Myositis and in Endoplasmic Reticulum Stress-Provoked Cultured Human Muscle Fibers. Am. J. Pathol. 177 (3), 1377–1387. 10.2353/ajpath.2010.100050 20616343 PMC2928970

[B32] Okazaki Y. Ohno H. Takase K. Ochiai T. Saito T. (2000). Cell Surface Expression of Calnexin, a Molecular Chaperone in the Endoplasmic Reticulum. J. Biol. Chem. 275 (46), 35751–35758. 10.1074/jbc.m007476200 10956670

[B33] Pinal-Fernandez I. Casal-Dominguez M. Huapaya J. A. Albayda J. Paik J. J. Johnson C. (2017). A Longitudinal Cohort Study of the Anti-synthetase Syndrome: Increased Severity of Interstitial Lung Disease in Black Patients and Patients with Anti-PL7 and Anti-PL12 Autoantibodies. Rheumatology (Oxford) 56 (6), 999–1007. 10.1093/rheumatology/kex021 28339994 PMC5850781

[B34] Preusse C. Allenbach Y. Hoffmann O. Goebel H. H. Pehl D. Radke J. (2016). Differential Roles of Hypoxia and Innate Immunity in Juvenile and Adult Dermatomyositis. Acta Neuropathol. Commun. 4 (1), 45. 10.1186/s40478-016-0308-5 27121733 PMC4847347

[B35] Preuße C. Goebel H. H. Held J. Wengert O. Scheibe F. Irlbacher K. (2012). Immune-Mediated Necrotizing Myopathy Is Characterized by a Specific Th1-M1 Polarized Immune Profile. Am. J. Pathol. 181 (6), 2161–2171. 10.1016/j.ajpath.2012.08.033 23058368

[B36] Rider L. G. Koziol D. Giannini E. H. Jain M. S. Smith M. R. Whitney-Mahoney K. (2010). Validation of Manual Muscle Testing and a Subset of Eight Muscles for Adult and Juvenile Idiopathic Inflammatory Myopathies. Arthritis Care Res. 62 (4), 465–472. 10.1002/acr.20035 PMC2924143 20391500

[B37] Rinnenthal J. L. Goebel H.-H. Preuße C. Lebenheim L. Schumann M. Moos V. (2014). Inflammatory Myopathy with Abundant Macrophages (IMAM): the Immunology Revisited. Neuromuscul. Disord. 24 (2), 151–155. 10.1016/j.nmd.2013.11.004 24314585

[B38] Salminen A. Kauppinen A. Suuronen T. Kaarniranta K. Ojala J. (2009). ER Stress in Alzheimer's Disease: a Novel Neuronal Trigger for Inflammation and Alzheimer's Pathology. J. Neuroinflammation 6, 41. 10.1186/1742-2094-6-41 20035627 PMC2806266

[B39] Seto N. Torres-Ruiz J. J. Carmona-Rivera C. Pinal-Fernandez I. Pak K. Purmalek M. M. (2020). Neutrophil Dysregulation Is Pathogenic in Idiopathic Inflammatory Myopathies. Jci Insight 5 (3). 10.1172/jci.insight.134189 PMC7098779 31945019

[B40] Song S. Tan J. Miao Y. Li M. Zhang Q. (2017). Crosstalk of Autophagy and Apoptosis: Involvement of the Dual Role of Autophagy under ER Stress. J. Cel Physiol 232 (11), 2977–2984. 10.1002/jcp.25785 28067409

[B41] Stengel S. T. Fazio A. Lipinski S. Jahn M. T. Aden K. Ito G. (2020). Activating Transcription Factor 6 Mediates Inflammatory Signals in Intestinal Epithelial Cells upon Endoplasmic Reticulum Stress. Gastroenterology 159 (4), 1357–1374. e10. 10.1053/j.gastro.2020.06.088 32673694 PMC7923714

[B42] Tanboon J. Uruha A. Stenzel W. Nishino I. (2020). Where Are We Moving in the Classification of Idiopathic Inflammatory Myopathies? Curr. Opin. Neurol. 33 (5), 590–603. 10.1097/wco.0000000000000855 32852298

[B43] Wang Q. Li Y. Ji S. Feng F. Bu B. (2018). Immunopathological Characterization of Muscle Biopsy Samples from Immune-Mediated Necrotizing Myopathy Patients. Med. Sci. Monit. 24, 2189–2196. 10.12659/msm.907380 29649184 PMC5914276

[B44] Wedderburn L. R. Varsani H. Li C. K. C. Newton K. R. Amato A. A. Banwell B. (2007). International Consensus on a Proposed Score System for Muscle Biopsy Evaluation in Patients with Juvenile Dermatomyositis: a Tool for Potential Use in Clinical Trials. Arthritis Rheum. 57 (7), 1192–1201. 10.1002/art.23012 17907237

[B45] Yasin S. A. Schutz P. W. Deakin C. T. Sag E. Varsani H. Simou S. (2018). Histological Heterogeneity in a Large Clinical Cohort of Juvenile Idiopathic Inflammatory Myopathy: Analysis by Myositis Autoantibody and Pathological Features. Neuropathol. Appl. Neurobiol. 45 (5), 495–512. 10.1111/nan.12528 PMC6767402 30378704

