# Supplementary material for: Endoplasmic Reticulum Stress Is Involved in Muscular Pathogenesis in Idiopathic Inflammatory Myopathies
Source: Front Cell Dev Biol. 2022 Feb 14;10:791986. doi: 10.3389/fcell.2022.791986 (PMC8882762; doi:10.3389/fcell.2022.791986)
Supplement: Supplementary file 1 [file Table1.DOCX]

**Supplementary Table 1.** Primers used for RT-qPCR assay

| Gene | Forward primer (5’→3’) | Reverse primer (3’→5’) |
| --- | --- | --- |
| SQSTM1 | TGCCTTGTACCCACATCTCC | AGCCGCTCCGATGTCATAGT |
| GAPDH | GAGTCCACTGGCGTCTTCAC | ATGACGAACATGGGGGCATC |
| GRP78 | ACGGGCAAAGATGTCAGGAA | CACTTTCTGGACGGGCTTCA |
| ATG2B | ATCGACATGCAACAAGGCAC | GGCACACCTGTCATTGCATC |
| ATG5 | ATAAGGCAACTGGGCTGGTC | AGACCTTCAGTGGTCCGGTA |
| BNIP3 | TATGGGATTGGTCAAGTCGGC | CATCAAAAGGTGCTGGTGGAG |
| PINK1 | CGTGAGACAGTTGGTGAGGG | GAGCCAGCCAACCATCTTGT |
| Beclin-1 | AACCAGATGCGTTATGCCCA | TCCATTCCACGGGAACACTG |
| CHOP | GGAGCTGGAAGCCTGGTATG | AAGCAGGGTCAAGAGTGGTG |
| XBP1 | GTCCGCAGCACTCAGACTAC | GGCTGGTAAGGAACTGGGTC |
| EDEM1 | GGGGACCCTTCAAATCTGAACAT | CGGCTTTCTGGAACTCGGAT |
