# Supplementary figures and images for: Endoplasmic Reticulum Stress Is Involved in Muscular Pathogenesis in Idiopathic Inflammatory Myopathies
Source: Front Cell Dev Biol. 2022 Feb 14;10:791986. doi: 10.3389/fcell.2022.791986 (PMC8882762; doi:10.3389/fcell.2022.791986)

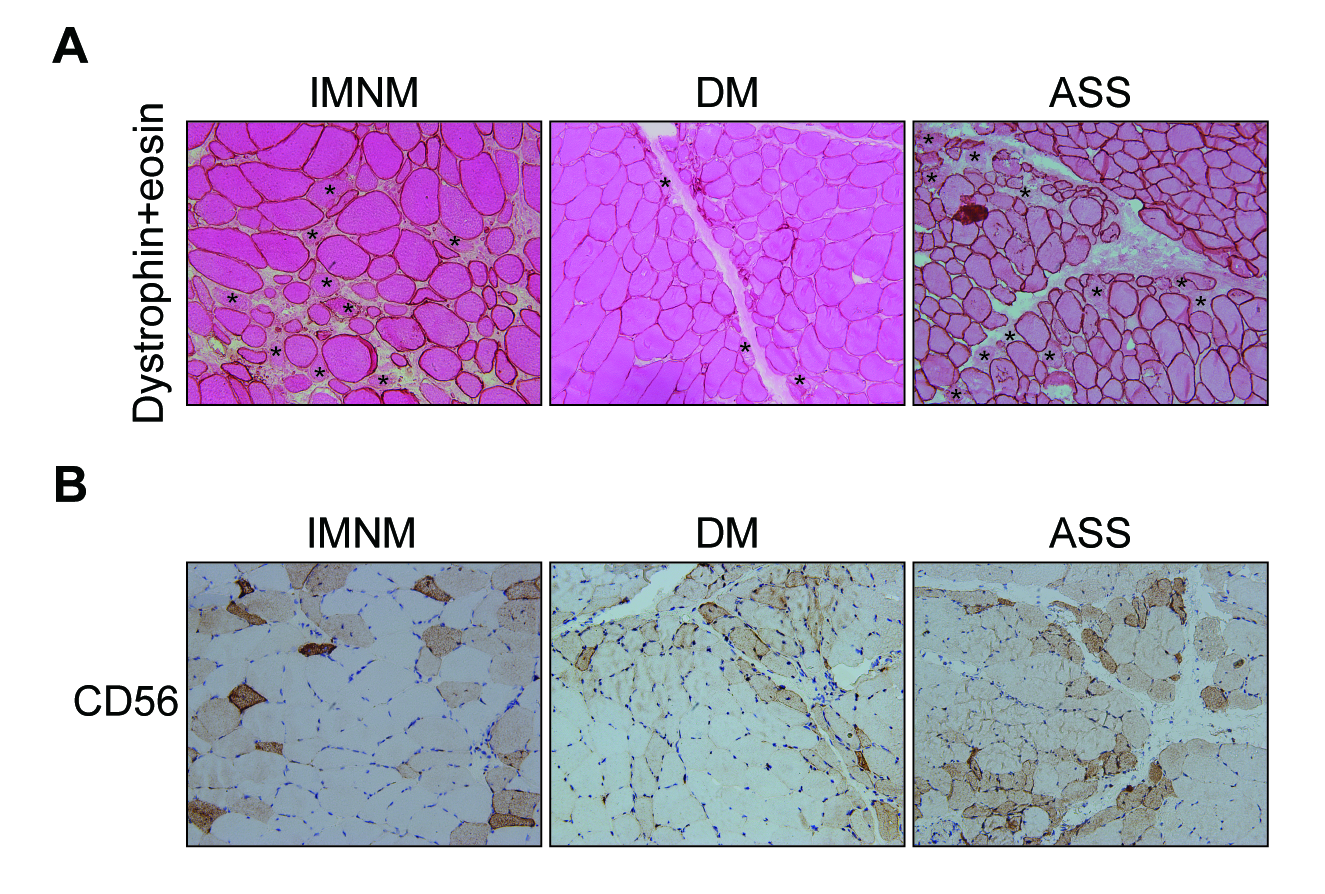

Supplement: Supplementary file 2 [file Image2.TIF]

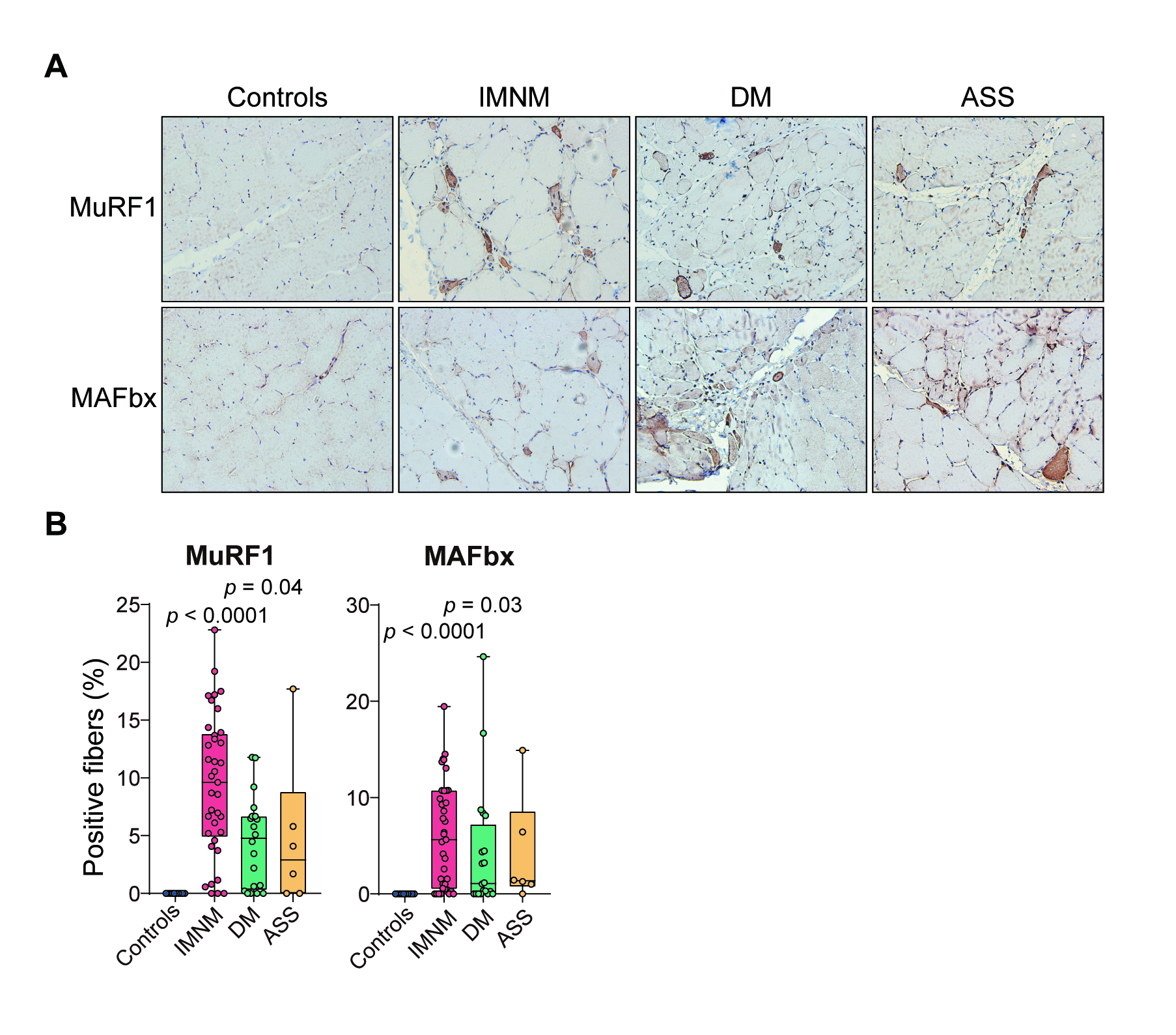

Supplement: Supplementary file 3 [file Image1.TIF]
